# Pathogen reduction through additive-free short-wave UV light irradiation retains the optimal efficacy of human platelet lysate for the expansion of human bone marrow mesenchymal stem cells

**DOI:** 10.1371/journal.pone.0181406

**Published:** 2017-08-01

**Authors:** Sabrina Viau, Lucie Chabrand, Sandy Eap, Judith Lorant, Karl Rouger, Francis Goudaliez, Chryslain Sumian, Bruno Delorme

**Affiliations:** 1 Biotherapy Division, Macopharma, Mouvaux, France; 2 INRA, UMR 703 PAnTher, Nantes, France; 3 LUNAM Université, Oniris, École Nationale Veterinaire, Agro-alimentaire et de L’alimentation Nantes-Atlantique, Nantes, France; 4 Transfusion Division, Macopharma, Tourcoing, France; University Hospital Modena and Reggio Emilia, ITALY

## Abstract

**Background:**

We recently developed and characterized a standardized and clinical grade human Platelet Lysate (hPL) that constitutes an advantageous substitute for fetal bovine serum (FBS) for human mesenchymal stem cell (hMSC) expansion required in cell therapy procedures, avoiding xenogenic risks (virological and immunological) and ethical issues. Because of the progressive use of pathogen-reduced (PR) labile blood components, and the requirement of ensuring the viral safety of raw materials for cell therapy products, we evaluated the impact of the novel procedure known as THERAFLEX UV-Platelets for pathogen reduction on hPL quality (growth factors content) and efficacy (as a medium supplement for hMSC expansion). This technology is based on short-wave ultraviolet light (UV-C) that induces non-reversible damages in DNA and RNA of pathogens while preserving protein structures and functions, and has the main advantage of not needing the addition of any photosensitizing additives (that might secondarily interfere with hMSCs).

**Methodology / Principal findings:**

We applied the THERAFLEX UV-Platelets procedure on fresh platelet concentrates (PCs) suspended in platelet additive solution and prepared hPL from these treated PCs. We compared the quality and efficacy of PR-hPL with the corresponding non-PR ones. We found no impact on the content of five cytokines tested (EGF, bFGF, PDGF-AB, VEGF and IGF-1) but a significant decrease in TGF-ß1 (-21%, n = 11, *p<0*.*01*). We performed large-scale culture of hMSCs from bone marrow (BM) during three passages and showed that hPL or PR-hPL at 8% triggered comparable BM-hMSC proliferation as FBS at 10% plus bFGF. Moreover, after proliferation of hMSCs in an hPL- or PR-hPL-containing medium, their profile of membrane marker expression, their clonogenic potential and immunosuppressive properties were maintained, in comparison with BM-hMSCs cultured under FBS conditions. The potential to differentiate towards the adipogenic and osteogenic lineages of hMSCs cultured in parallel in the three conditions also remained identical.

**Conclusion / Significance:**

We demonstrated the feasibility of using UV-C-treated platelets to subsequently obtain pathogen-reduced hPL, while preserving its optimal quality and efficacy for hMSC expansion in cell therapy applications.

## Introduction

Human bone marrow-mesenchymal stem cells (BM-hMSCs) are adult multipotent stem cells with a potential for multi-lineage differentiation, a hematopoiesis-supportive capacity [1] and migration / homing properties [2]. In addition to these characteristics, their immunosuppressive properties [3, 4] and their profile of secretion of trophic factors [5] lead to an increase in hMSC uses in cell therapy clinical trials, mainly for immunomodulation and regenerative medicine purposes [6, 7].

In this context, defining proper conditions for necessary *ex vivo* hMSC expansion is critical. In particular, the immunological risk induced by the use of fetal bovine serum (FBS) as a medium supplement for cell culture in cell therapy procedures needs to be addressed [8]. Studies showed that FBS proteins may be internalized by hMSCs during culture (up to 7-30mg/cell), showing perinuclear localization [9]. Antibodies against FBS proteins may be detected in patient serum following cell infusion [10]. Human platelet lysate (hPL), obtained from the lysis of human platelets, is particularly rich in growth factors and nutritive elements and may constitute a non-xenogenic substitute for FBS [11, 12]. Indeed, the use of hPL and its derivatives in hMSC culture has been documented since 2005 [13] and 2003 [14], respectively, and hPL is commonly recognized as a way to avoid xenogenic risks (viruses and immunological) linked to FBS [15].

Although there are few reported cases of platelet transfusion-transmitted bacterial infections (estimated at 0.001% in the USA [16] and between 0.001% and 0.004% in Europe [17, 18]), the bacterial contamination of platelet concentrates (PCs) (estimated < 0.1% in Germany [19]) currently remains an issue in transfusions [20], mainly due to the storage of PCs at ambient temperature. The risk of bacterial contamination of hPL derived from PCs can be eliminated *via* a final step of aseptic filtration (pore size of 0.22 μm) in the production process. However, the issue of potential human virus contamination still remains, even if blood donors are screened and each blood collection is tested according to country specific regulations. The risk of viral transmission in transfusion appears to be very low for “well-known” viruses, such as HIV-1 and -2 (1 per 2.3 million blood product donations in USA) or hepatitis C (1 per 1.8 million) [15, 21] but cannot be completely excluded. Also of concern are (re)emerging viruses and variants of existing viruses. Furthermore, the species barrier is bypassed when FBS is replaced by hPL for human cell culture. Consequently, a viral inactivation step of hPL may rapidly become a mandatory regulatory requirement when *in vitro* expanded cell therapy products are used for clinical applications [22].

THERAFLEX UV-Platelets is a pathogen reduction technology for PCs based on ultraviolet (UV) light absorption by nucleic acids (DNA and RNA) [23]. This in turn causes the formation of cyclobutane pyrimidine and pyrimidine pyrimidone dimers, which block the elongation of nucleic acid transcripts [24]. Under orbital agitation, PCs are subjected to double-sided UV-C irradiation at a wavelength (254 nm) leading, on one side, to non-reversible damages in DNA and RNA of viruses, bacteria and parasites while, on the other side, preserving protein structures and functions [25]. The efficacy of this additive-free technology has been reported on lipid-enveloped and non-enveloped viruses [26], and a phase I clinical trial has been completed, demonstrating the safety and tolerability of THERAFLEX UV-Platelets-treated autologous PCs in subjects [27].

In this study, we evaluated the impact of the THERAFLEX UV-Platelets procedure for pathogen reduction of PCs used to produce hPL. The quality (growth factors content) and efficacy (as a medium supplement for hMSC proliferation) were assessed, and we particularly investigated the efficiency of hPL prepared from UV-C pathogen-reduced PCs for BM-hMSC expansion, while preserving their differentiation potential and immunosuppressive properties.

## Material & methods

### Pathogen reduction of PCs using THERAFLEX UV-Platelets

Leucoreduced PCs, obtained from pools of five buffy coats each, suspended in SSP+ additive solution (Macopharma, Mouvaux, France) and prepared according to French transfusion practices were obtained from EFS Nord de France. PCs were subjected to UV-C treatment using dedicated illumination devices (kit XUV4005XU and Macotronic UV illumination machine, Macopharma), in accordance with the THERAFLEX UV-Platelets procedure. A standard illumination dose of 0.2 J/cm^2^ was used under agitation of the bag, as recommended by the manufacturer. An aliquot of each PC was collected before illumination for non-irradiated control.

### HPL and PR-hPL preparation

Treated and non-treated PCs were frozen at -80°C and thawed overnight at +4°C. Centrifugation was performed at 3,500 g for 30 min, with low break. The pellet composed of cell debris was discarded. Platelet lysates prepared from non-irradiated (hPL) and irradiated PCs (PR-hPL) were aliquoted and stored at -80°C. One hPL unit and one PR-hPL unit were prepared from one PC. Before use for hMSC culture, hPL and PR-hPL were thawed at +37°C, and used individually (corresponding to five donors in each unit) or as batches of three units (corresponding to 15 donors in each batch).

### Growth factor assay in hPL and PR-hPL

The contents of basic fibroblast growth factor (bFGF, #DFB50), vascular endothelial growth factor (VEGF, #DVE00), epidermal growth factor (EGF, #DEG00), platelet-derived growth factor-AB (PDGF-AB, #DHD00C), insulin-growth factor (IGF)-1 (#DG100) and transforming growth factor (TGF)-ß1 (#DB100B) in PR-hPL and their respective hPL controls were measured by ELISA (Bio-techne, Minneapolis, USA), following manufacturer instructions. The absorbance was measured using an Infinite^®^ M200 PRO spectrometer (Tecan, Männedorf, Switzerland), and the results were analyzed using Magellan^™^ data analysis software (Tecan).

### BM-hMSC culture

BM-hMSCs were cultured as previously described [28]. Briefly, cells were seeded on a cell culture-treated surface (Corning, New York, USA) in the presence of Minimum Essential Medium (MEMα) manufactured under GMP conditions (Macopharma) and supplemented with either MSC-qualified FBS (Gibco, Life Technologies, Carlsbad, USA) with 1 ng/mL bFGF (Eurobio, Montpellier, France) or hPL or PR-hPL. Heparin (Biochrom, VWR, Radnor, USA) at 2 IU/mL was added to hPL- and PR-hPL-containing media to avoid gelation of the medium. 100 U/mL penicillin G / 0.1 mg/mL streptomycin sulfate (Lonza, Basel, Switzerland) was added under all conditions, and the media were renewed twice a week. Cell cultures were maintained in a humidified atmosphere containing 5% CO_2_. All experiments were performed between P1 and P4.

### BM-hMSC proliferation determination

For miniaturized cell proliferation experiments, cells were seeded at 3,000 cells/cm^2^ in 96-well plates (Corning). BM-hMSCs were cultured for 10 days under the culture conditions described above, with the concentration of FBS, hPL or PR-hPL ranging from 2 to 15%. Cell proliferation was determined using the CellTiter-Glo luminescent kit (Promega Corporation, Madison, USA) in accordance with the manufacturer’s instructions. The luminescence level was measured using an Infinite^®^ M200 PRO spectrometer (Tecan) and analyzed using i-control^™^ software (Tecan).

For “large-scale” BM-hMSC proliferation, cells were seeded at 4,000 cells/cm^2^ in 75 cm^2^ flasks (Corning). Cells were cultured using the culture conditions described above, with FBS at 10% v/v + 1 ng/mL bFGF or with hPL or PR-hPL at 8% v/v. When the cell layer under hPL conditions reached confluency, the medium was discarded, the cell layer was washed twice with phosphate buffer saline (PBS, Macopharma) and dissociated with TrypLE^™^ (Gibco). Cells were centrifuged at 300 g for 5 min and diluted in 10 mL of the appropriate medium. Cell number was determined using a cell counter, and viability was assessed by Trypan Blue exclusion (ViCell XR, Beckman Coulter, Brea, USA).

### Determination of BM-hMSC clonogenic potential

After proliferation of BM-hMSCs under the different culture conditions tested, the colony-forming unit-fibroblast (CFU-F) assay was performed as previously described [28]. After cell layer dissociation by TrypLE^™^ (see above), cells were seeded at 100 and 200 cells in 25 cm^2^ flasks (Corning). The medium was renewed on days 3 and 7. After 12 days of culture, cell layer was washed twice with PBS, fixed with 4% paraformaldehyde (PFA, Sigma-Aldrich, St Louis, USA) in PBS for 10 min and washed twice with PBS. Colonies were stained with May-Grünwald (RAL, VWR), washed twice with distilled water and counterstained with Giemsa (Merck, VWR) diluted 10 times in PBS. Giemsa was then removed, and colonies were finally washed twice with water. Individual colonies composed of at least 50 cells were counted. CFU-F frequency was calculated based on the respective input cell number as numbers of CFU-F / BM-hMSCs initially plated (in percentage).

### BM-hMSC immunophenotype

After proliferation of BM-hMSCs under the different culture conditions tested, the expression of a panel of surface markers was assessed, following previously described protocols [28]. After cell layer dissociation by TrypLE^™^ (see above), BM-hMSCs were subjected to centrifugation at 350 g for 5 min. Cells were resuspended in cold PBS and then centrifuged at 350 g for 5 min. For each antigen tested, 200,000 cells resuspended in cold PBS were incubated with phycoerythrin (PE)-conjugated CD29 (#555443), CD34 (#345802), CD40 (#555589), CD45 (#555483), CD73 (#550257), CD80 (#PN IM1976U), CD86 (#PN IM2729U), CD90 (#555596), CD105 (#560839) or HLA-DR (#PN IM0464U) monoclonal antibody, at saturating concentration, for 30 min in the dark at +4°C. Appropriate PE-conjugated isotype-matched controls (mouse IgG1 #555749 and IgG2b #555743) were included. Antibodies and isotype controls were purchased from Becton-Dickinson (Durham, USA; CD29, CD34, CD40, CD45, CD73, CD90, CD105, IgG1 and IgG2b) or Beckman Coulter (CD80, CD86 and HLA-DR). BM-hMSCs were then washed twice with PBS by centrifugation at 350 g for 5 min. Pellets of BM-hMSCs were finally resuspended in 200 μL of CellFix (Becton-Dickinson) and processed immediately for flow cytometric analysis. Acquisitions were performed using an ACCURI^™^C6 flow cytometer equipped with 488 nm argon laser (Becton-Dickinson). At least 10,000 events were recorded for each analysis.

### Differentiation potential assay of BM-hMSCs

After proliferation of BM-hMSCs in the different culture conditions tested, their adipogenic and osteogenic differentiation capacity was assessed by seeding cells at 30,000 cells/cm^2^ on an appropriate surface: 12-well plates, 96-well black with clear bottom plates (Corning) or glass slides. Previously described protocols [28] were followed. Adipogenic differentiation was induced using Dulbecco’s modified Eagle’s medium (DMEM) low glucose (#31885, Gibco) supplemented with 10% FBS, 1 μM dexamethasone (Sigma-Aldrich), 0.5 mM 3-isobutyl-1-methylxanthine (Sigma-Aldrich) and 60 μM indomethacine (Sigma-Aldrich). Osteogenic differentiation was induced using DMEM high glucose (#41965, Gibco) supplemented with 10% FBS, 0.1 μM dexamethasone, 25 μg/mL L-ascorbic acid (Sigma-Aldrich) and 3 mM NaH_2_PO_4_ (Sigma-Aldrich). Antibiotics (see above) were added to the cell culture medium, and the medium was renewed twice a week.

After 14 days of culture, the adipogenic differentiation was revealed using Oil Red O and Nile Red stainings after fixation with 4% PFA (see above). Briefly, the cell layer was stained with 1.8 g/L Oil Red O (Sigma-Aldrich) for 30 min. Lipid droplets in the cytoplasm of the cells appeared to be stained in red. For Nile Red staining, the cell layer was incubated with 1 μg/mL Nile Red (Sigma-Aldrich) for 30 min in the dark at +4°C and counterstained with DAPI (Vectashield^™^, VWR) to visualize the nuclei. The wavelengths used were: 480 nm/527 nm for Nile Red and 360 nm/470 nm for DAPI (excitation/emission). Nile Red positive and negative cells were independently counted by two operators. For each condition, a minimum of 550 cells were counted on a minimum of 23 photographs. The ratio of numbers of positive cells to total cells was calculated. The accumulation of triglycerides was quantified with a commercially available kit according to the manufacturer’s instructions. Briefly, the cell layer was washed with PBS and incubated with the AdipoRed^™^ reagent (Lonza) for 10 min. The fluorescence was measured using an Infinite^®^ M200 PRO spectrometer (Tecan). The parameters used were: excitation wavelength at 485 nm, emission wavelength at 572 nm and gain at 90. The results were analyzed using Magellan^™^ data analysis software (Tecan).

After 21 days of culture, the osteogenic differentiation was revealed using Alizarin Red S and Von Kossa stainings after fixation with 4% PFA (see above). Briefly, the cell layer was stained with 2% Alizarin Red S at pH 4.3 (Sigma-Aldrich) for 30 sec to 5 min. The staining reaction was stopped with distilled water. Calcium deposits appeared to be stained in red-orange. For the Von Kossa staining, the cell layer was stained with 4% AgNO_3_ (Sigma-Aldrich) for 30 min in the dark. The cell layer was then washed twice with distilled water, covered with distilled water and exposed to light for 1 hour. The staining reaction was stopped with 5% thiosulfate (Sigma-Aldrich) for 2 min. The extracellular matrix appeared to be stained in black. For both stainings, the total area of the wells was pictured and an analysis using the ImageJ software selectively quantified positively stained areas. For each condition, a minimum of 19cm^2^ was analyzed. ALP activity measurement was performed with commercially available kits (Interchim, Montluçon, France and Abcam, Cambridge, United Kingdom, respectively), in accordance with the manufacturer’s instructions. ALP activity level was normalized with the protein concentration measured using the BCA technique, in accordance with the manufacturer’s instructions.

### Evaluation of immunosuppressive properties of BM-hMSCs

Human CD3+ T-cells were obtained from whole blood samples of donors (Clinical Transfer Facility, CICBT0503, Nantes, France) by centrifugal counter-flow elutriation and isolation by negative magnetic sorting using an EasySep^™^ kit (>90% purity; Stemcell Technologies, Vancouver, Canada).

After proliferation of BM-hMSCs under the different culture conditions tested, their ability to suppress proliferation of T-cells stimulated with concavaline A (Con A, Sigma-Aldrich) was assessed. The cell layer was dissociated (see above) and irradiated at 35 Gy for 10 min. Recovered BM-hMSCs (5,000, 10,000, 20,000 or 100,000 cells per well) were co-cultured for five days with T-cells (100,000 per well) activated with Con A (10 μg/mL) in 96-well plates (BM-hMSC:T-cell ratios: 1:20, 1:10, 1:5 and 1:1). Controls included non-activated and activated T-cells with no BM-hMSCs.

After proliferation of BM-hMSCs under the different culture conditions tested, their ability to suppress proliferation of T-cells stimulated with allogeneic irradiated (35 Gy) PBMCs (MLR assay) was assessed. The cell layer was dissociated (see above) and irradiated at 35 Gy for 10 min. Recovered BM-hMSCs (20,000 or 100,000 cells per well) were co-cultured for five days with T-cells (100,000 per well) activated with PBMCs (100,000 cells per well) in 96-well plates (BM-hMSC:T-cell:PBMC ratios: 1:5:5 and 1:1:1). Controls included non-activated and activated T-cells with no BM-hMSCs.

For both experiments, cells were then incubated overnight with tritiated thymidine (0.925 μBq/mL, PerkinElmer, Zaventem, Belgium) and harvested on a filter using Harvester Mach III (Tomtec, Hamden, USA). Radioactivity was measured on the filters using 1450 MicroBeta Jet (Perkin Elmer). Percentages of inhibition were calculated as follows:

% inhibition = 100 − radioactivity measurement of the sample / radioactivity measurement of activated T-cell control.

### Statistical analysis

Statistical analyses were performed using PRISM Software. Student’s t-test, one-way ANOVA or two-way ANOVA and Bonferroni posttests were applied when appropriate. P<0.05 was considered statistically significant.

## Results

### UV-C illumination of PCs induced a slight decrease in TGF-ß1 content in hPL

**Fig 1:**
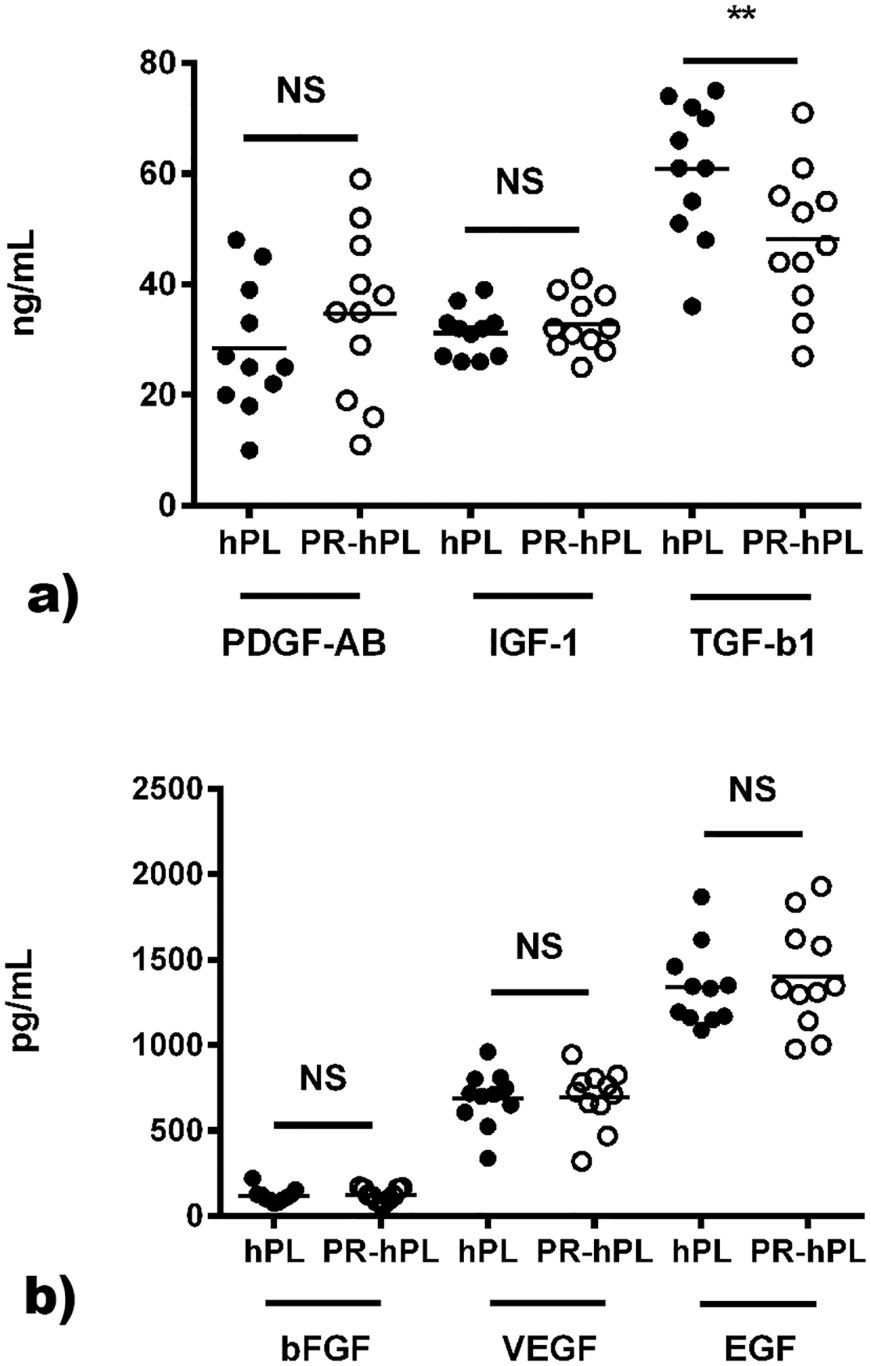
Growth factor contents in hPL and PR-hPL measured using commercially available ELISA kits. Results are presented as concentrations of PDGF-AB, IGF-1, TGF-ß1 (**a**), and bFGF, VEGF and EGF (**b**), (individual values and means of dosages in n = 11 units of PR-hPL and their respective hPL controls). **: *p<0*.*01* hPL *versus* PR-hPL (Student’s t-test).

We determined the concentration of bFGF, VEGF, EGF, PDGF-AB, IGF-1 and TGF-ß1 in 11 units of PR-hPL and their respective hPL controls (Fig 1). We did not find any significant effect of the UV-C illumination of PCs on bFGF, VEGF, EGF, PDGF-AB and IGF-1 contents in hPL. Under both conditions, the major growth factors present were IGF-1, PDGF-AB and TGF-ß1. In hPL, IGF-1 varied from 26.5 ng/mL to 38.6 ng/mL, PDGF-AB from 9.66 ng/mL to 47.9 ng/mL and TGF-ß1 from 35.9 ng/mL to 74.5 ng/mL. The contents of bFGF, VEGF and EGF were at least ten-fold lower, without any significant difference between hPL and PR-hPL. In hPL, bFGF varied from 75.8 pg/mL to 221 pg/mL, VEGF from 338 pg/mL to 961 pg/mL and EGF from 1,089 pg/mL to 1,868 pg/mL. Interestingly, TGF-ß1 was found to be slightly but significantly affected by UV-C illumination, with a decrease of 21% (from 61 ± 12 ng/mL to 48 ± 13 ng/mL, *p<0*.*01*).

### hPL or PR-hPL triggered similar proliferation of BM-hMSCs

The possibility to use PR-hPL as a cell culture supplement for BM-hMSC proliferation was evaluated.

**Fig 2:**
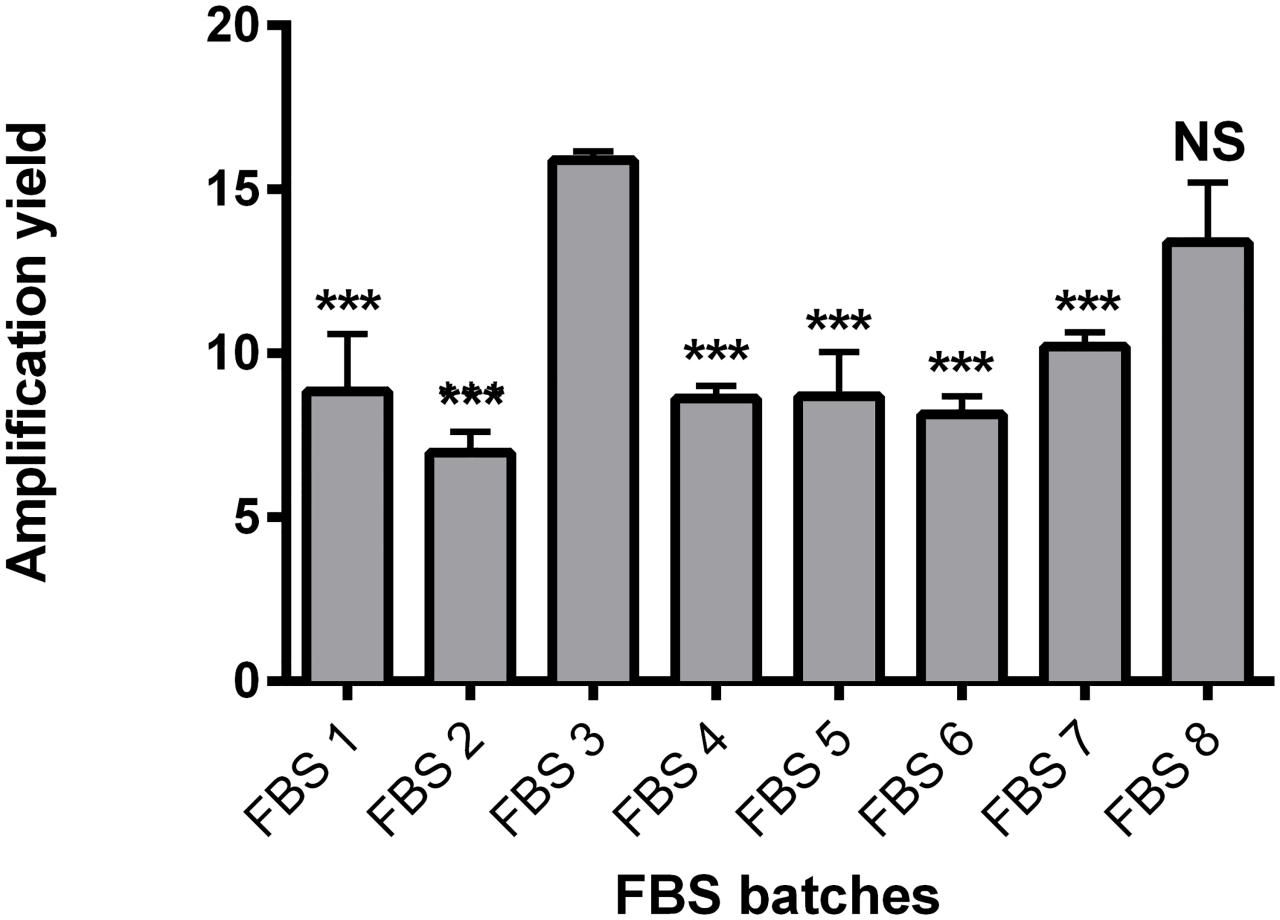
Proliferation of BM-hMSCs cultured in an FBS-containing medium. Eight references of FBS were tested at a dose of 10% with 1 ng/mL bFGF. Results are presented as amplification yields for means of triplicates. NS: *not significant*; ***: *p<0*.*001 versus* FBS 3 (one-way ANOVA and Bonferroni posttests).

In a first step, because FBS displays batch-to-batch variability, requiring batch screening for hMSC culture, we carefully screened and selected an efficient FBS batch that we subsequently used as our control. Eight references of FBS were compared when used as a supplement for BM-hMSC proliferation, at the typical dose of 10% with bFGF at 1 ng/mL (Fig 2). Our results highlighted that huge variations were observed, the amplification yield ranging from 6.98 ± 0.63 to 15.88 ± 0.27. FBS batch 3 leads to the best proliferation of BM-hMSCs (significant difference from all the other FBS batches except FBS batch 8) and is hMSC-dedicated from the manufacturer. We decided to select this batch of FBS for the following experiments.

We also performed additional experiments to evaluate the impact of the heparin concentration on BM-hMSC proliferation (S1 Fig). We first tested heparin at doses ranging from 0 to 64 IU/mL in a 10% FBS + 1ng/mL-containing medium. We showed that there was no impact until 2 IU/mL. From 4 IU/mL, the heparin addition significantly impaired BM-hMSC proliferation. We then tested heparin at doses ranging from 1 to 64IU/mL in an 8% hPL-containing medium and observed that heparin at 2IU/mL did not impair BM-hMSC proliferation. A dose of 4IU/mL resulted in a decrease in cell proliferation that became significant from 8IU/mL. A dose of 2IU/mL of heparin was used for the following experiments.

**Fig 3:**
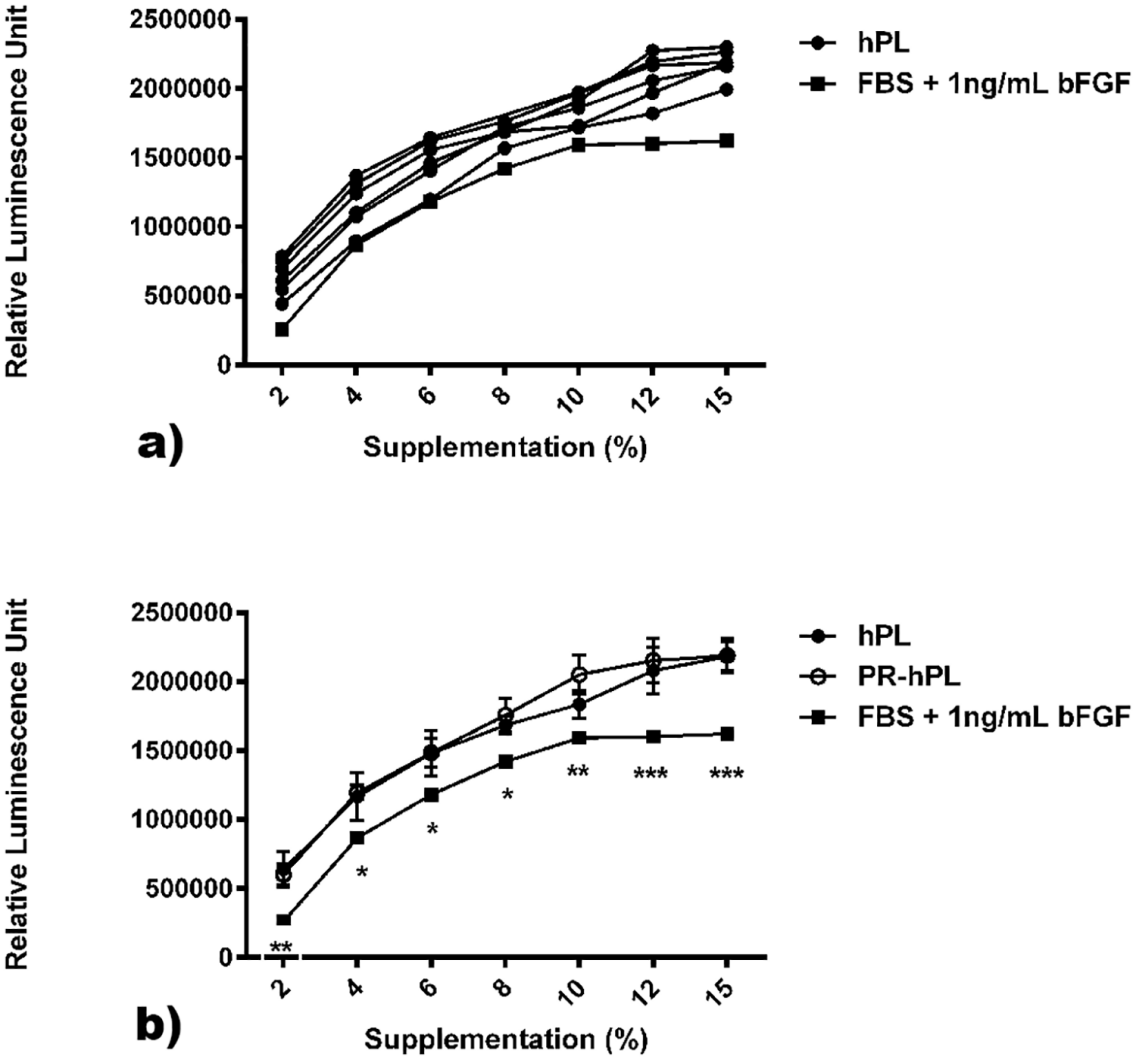
Proliferation of BM-hMSCs cultured for 10 days in an FBS+bFGF-, hPL- or PR-hPL-containing medium. Six units of PR-hPL and their respective hPL controls were tested at doses ranging from 0% to 15%. FBS (from 2% to 15%) with 1 ng/mL bFGF was used as a control. Proliferation was evaluated using the CellTiter-Glo assay. *: *p<0*.*05*; **: *p<0*.*01*; *** *p<0*.*001 versus* hPL / PR-hPL (two-way ANOVA and Bonferroni posttests).

In a second step, we cultured BM-hMSCs for 10 days in a medium containing hPL or PR-hPL (at doses ranging from 0% to 15%) or FBS (from 2% to 15%, with bFGF at 1 ng/mL) (Fig 3). Individual units of hPL or PR-hPL were used. We could observe a dose-effect of hPL, PR-hPL and FBS on cell proliferation (Fig 3a and 3b). The variability between units of hPL was minimal (1.12-fold) and maximal (1.77-fold) at the doses of 8% and 2%, respectively (Fig 3a). When compared to the FBS-containing medium, the hPL-containing medium was always better or equivalent, considering individual units (Fig 3a) or a mean of six units (Fig 3b). Moreover, a dose of 8% hPL always presented a better or comparable efficacy than a dose of 10% FBS with 1 ng/mL bFGF (typical dose for BM-hMSC proliferation) (Fig 3a and 3b). Most interestingly, UV-C illumination of PCs appeared to have no effect on hPL efficacy (Fig 3b). Whatever the dose of supplementation, we never observed differences between hPL and PR-hPL, even in stringent cell culture conditions far below the confluency stage (doses of supplementation as low as 2% and 4%). We showed that PR-hPL was still more efficient than FBS (Fig 3b). Finally, we could observe that for the 10% dose, BM-hMSCs proliferation seemed to reach a maximum under FBS with bFGF conditions, while it may be further increased with higher supplementation under hPL or PR-hPL conditions, suggesting that the difference in efficacy between FBS with bFGF and hPL or PR-hPL may be further increased.

Considering these first results, we chose the 8% dose of hPL or PR-hPL for further investigations. Batches of PR-hPL and hPL were obtained by pooling three units of PR-hPL and pooling the three respective hPL controls. Thus, each batch of hPL or PR-hPL included 15 donors. 10% FBS with bFGF at 1 ng/mL was used as a control.

**Fig 4:**
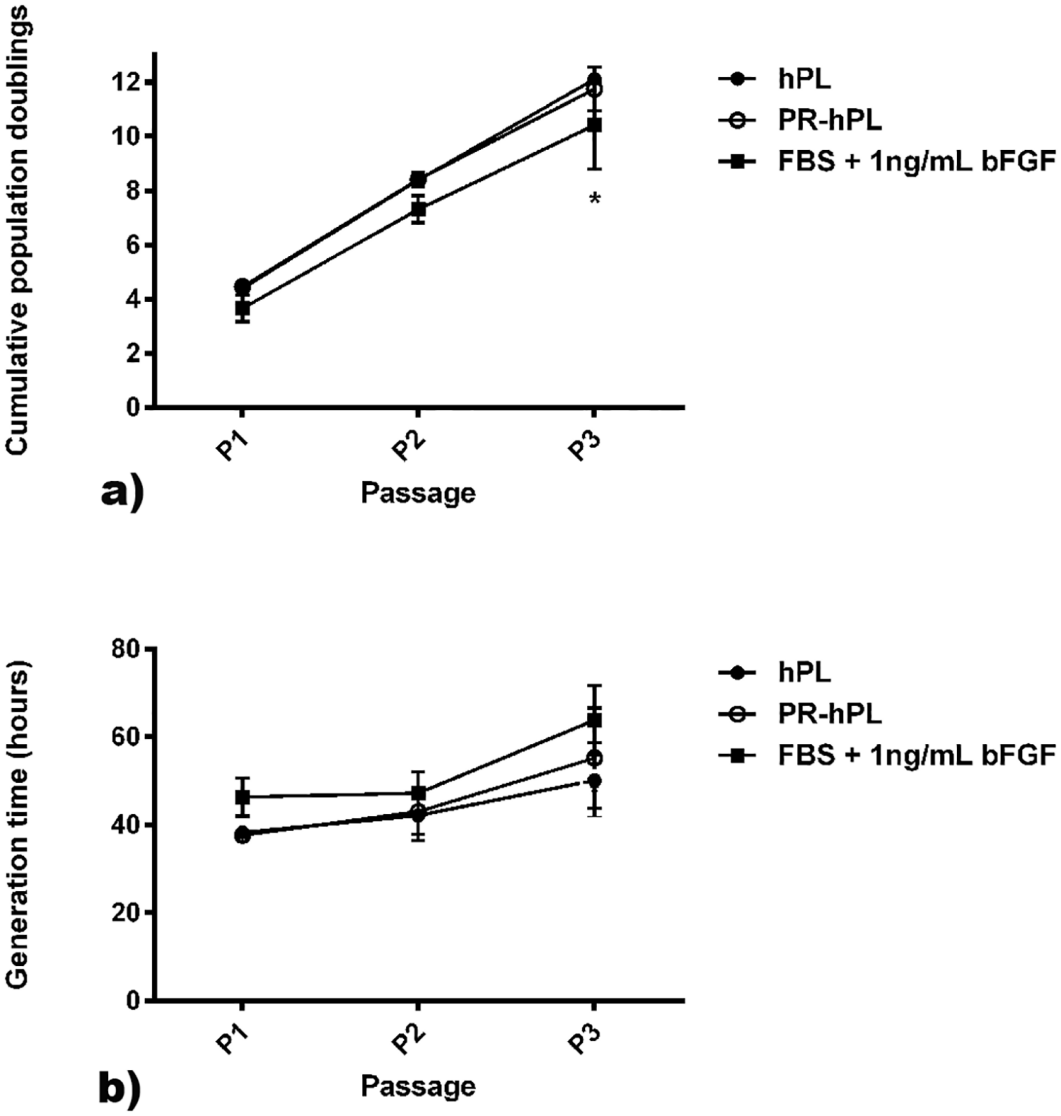
Proliferation of BM-hMSCs cultured in an FBS+bFGF-, hPL- or PR-hPL-containing medium during three consecutive passages. Results are presented as cumulative population doubling (**a**) and generation time (**b**), for means of n = 3 experiments. *: *p<0*.*05 versus* hPL / PR-hPL (two-way ANOVA and Bonferroni posttests).

In a third step, we compared the proliferation of BM-hMSCs cultured during three consecutive passages under the three different medium conditions: 10% FBS + 1 ng/mL bFGF, 8% hPL and 8% PR-hPL (Fig 4). Our results confirmed that using 8% hPL or PR-hPL leads to comparable proliferation of cells compared to 10% FBS + bFGF for the first and second passages (Fig 4a). After the third passage, the cumulative population doubling in the hPL- or PR-hPL-containing medium was significantly higher than in FBS-containing medium (Fig 4a, 12.1 ± 0.3 or 11.7 ± 0.8 *versus* 10.4 ± 1.6, *p<0*.*05*). The generation time was decreased in the hPL- or PR-hPL-containing medium, in comparison with the FBS-containing medium (Fig 4b, not significant). The generation time increased in passage 2 *versus* passage 1 and in passage 3 *versus* passage 2, whatever the culture conditions (Fig 4b, overall effect of the passage: *p<0*.*001*). Our results showed no differences between PR-hPL and hPL (Fig 4a and 4b).

### hPL- or PR-hPL-containing medium preserved the clonogenic potential of BM-hMSCs

We also verified that the clonogenic potential of BM-hMSCs was maintained after culture under the different conditions. We showed no differences in the number of CFU-F between BM-hMSCs previously cultured in the hPL- and PR-hPL-containing medium: 23.7% ± 8.4% *versus* 22.9% ± 4.7%, respectively (n = 3 experiments, not significant).

### Culture in hPL or PR-hPL did not alter membrane marker expression of BM-hMSCs

**Fig 5:**
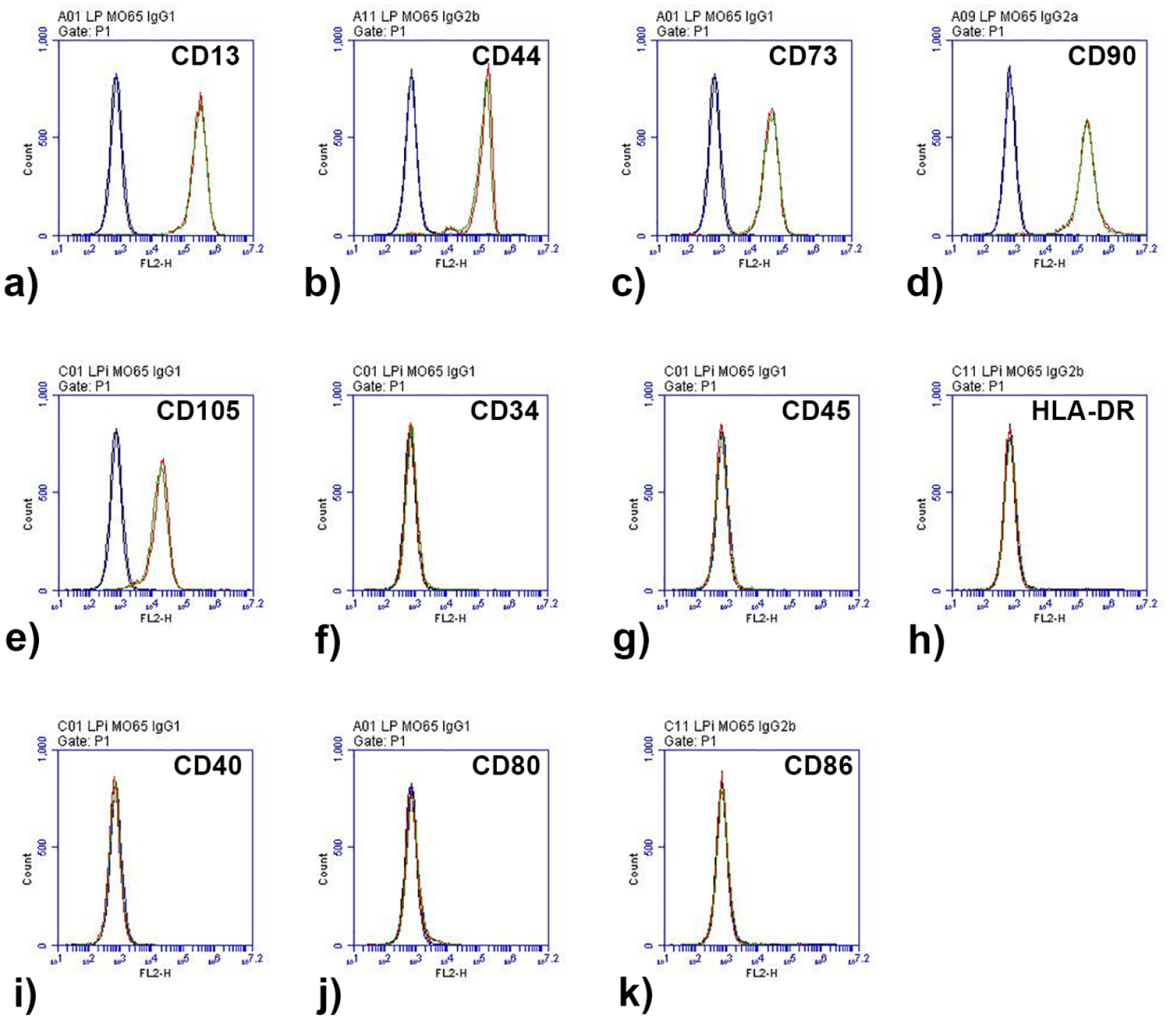
Representative histogram overlays for expression of CD13 (a), CD44 (b), CD73 (c), CD90 (d), CD105 (e), CD34 (f), CD45 (g), HLA-DR (h), CD40 (i), CD80 (j) and CD86 (k) of BM-hMSCs cultured in an hPL- (red curves *versus* isotype controls in black) or PR-hPL-containing medium (green curves *versus* isotype controls in blue).

We investigated membrane marker expression of BM-hMSCs after proliferation in the FBS+bFGF-, hPL-, or PR-hPL-containing medium (Table 1 and Fig 5), in accordance with ISCT guidelines [29, 30]. Our results showed that cells express BM-hMSC membrane markers (CD13, CD44, CD73, CD90 and CD105), whatever the culture conditions, and did not express hematopoietic markers (CD34 and CD45) or major histocompatibility complex class II (HLA-DR). We also verified that culture conditions did not induce the expression of co-stimulatory molecules. BM-hMSCs were found negative for the expression of CD40, CD80 and CD86 markers, whatever the culture conditions. We found that neither hPL nor PR-hPL impaired membrane marker expression of BM-hMSCs.

**Table 1:** Expression of CD13, CD34, CD40, CD44, CD45, CD73, CD80, CD86, CD90, CD105 and HLA-DR in BM-hMSCs cultured in an FBS+bFGF-, hPL- or PR-hPL-containing medium assessed by flow cytometry. Results are presented as percentages of positive cells for means of experiments with hMSCs from n = 3 BM.

% of positive cells	10% FBS + 1 ng/mL bFGF	8% hPL	8% PR-hPL
BM-hMSC markers			
**CD13**	99.8 ± 0.2	99.9 ± 0.1	99.7 ± 0.4
**CD44**	96.9 ± 2.7	98.3 ± 0.9	98.8 ± 2.0
**CD73**	93.1 ± 0.3	98.6 ± 0.1	98.8 ± 0.3
**CD90**	97.8 ± 2.3	99.5 ± 0.3	99.3 ± 1.1
**CD105**	85.2 ± 2.8	92.1 ± 1.5	91.0 ± 1.2
Hematopoietic markers			
**CD34**	0.33 ± 0.10	0.10 ± 0.06	0.17 ± 0.12
**CD45**	0.03 ± 0.02	0.04 ± 0.02	1.06 ± 1.65
Major Histocompatibility Complex class II			
**HLA-DR**	0.81 ± 0.67	0.22 ± 0.12	0.03 ± 0.03
Co-stimulatory molecules			
**CD40**	0.01 ± 0.01	0.01 ± 0.02	0.48 ± 0.77
**CD80**	1.33 ± 0.13	1.02 ± 0.68	1.18 ± 0.79
**CD86**	0.12 ± 0.16	0.49 ± 0.49	0.07 ± 0.12

### BM-hMSC differentiation potential was maintained using hPL or PR-hPL in the culture medium

**Fig 6:**
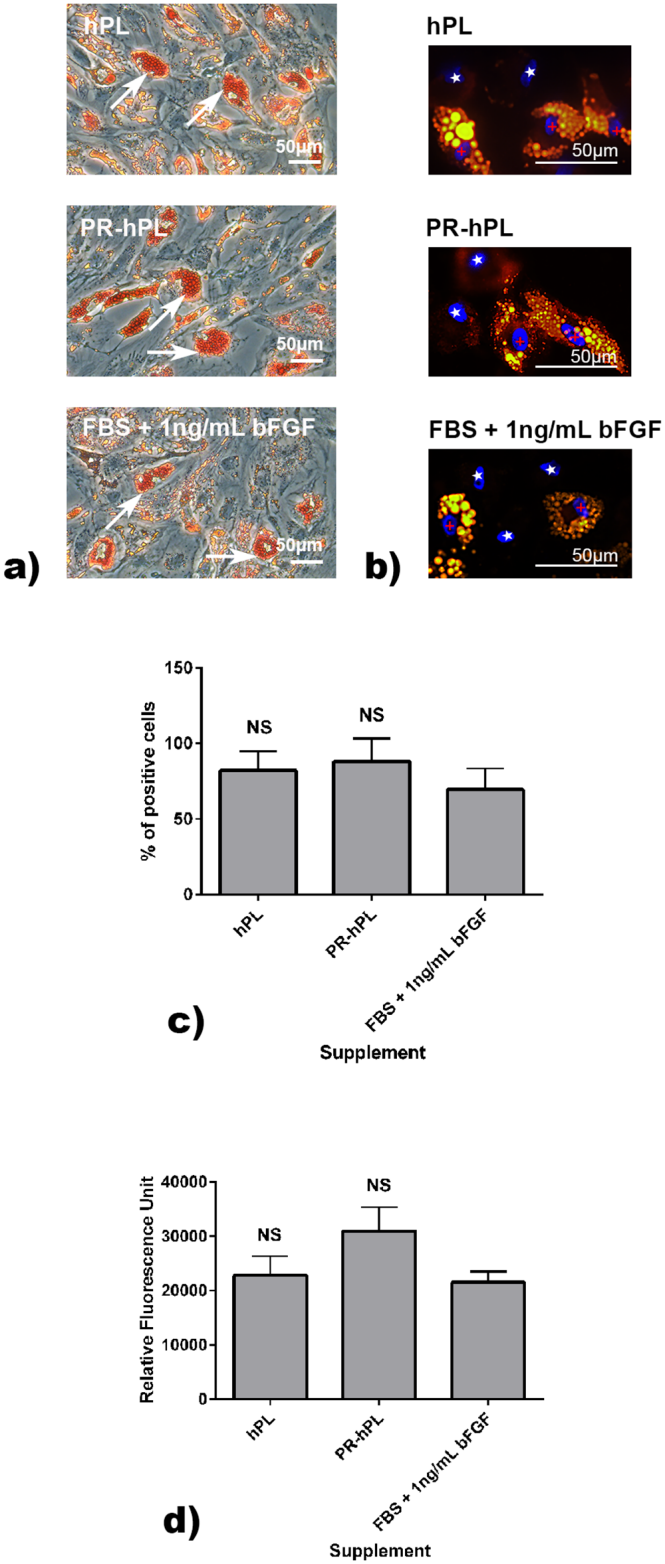
Adipocyte differentiation potential of BM-hMSCs after culture in an FBS+bFGF-, hPL- or PR-hPL-containing medium. Differentiation was induced using the specific medium. Lipid droplets in adipocytes were stained using Oil Red O (**a**) or Nile Red (**b**). Representative photographs of experiments with hMSCs from n = 3 BM. White arrows illustrated Oil Red O positively stained lipid vesicles. Cells positive for DAPI and Nile Red were indicated with red crosses and cells positive for DAPI but negative for Nile Red with white stars. Quantification of Nile Red was expressed as a percentage of positive cells (**c**).NS: *not significant versus* FBS. The accumulation of triglycerides was evaluated using a commercially available kit (**d**, means of 18 wells for each condition and each time point). NS: *not significant versus FBS*.

**Fig 7:**
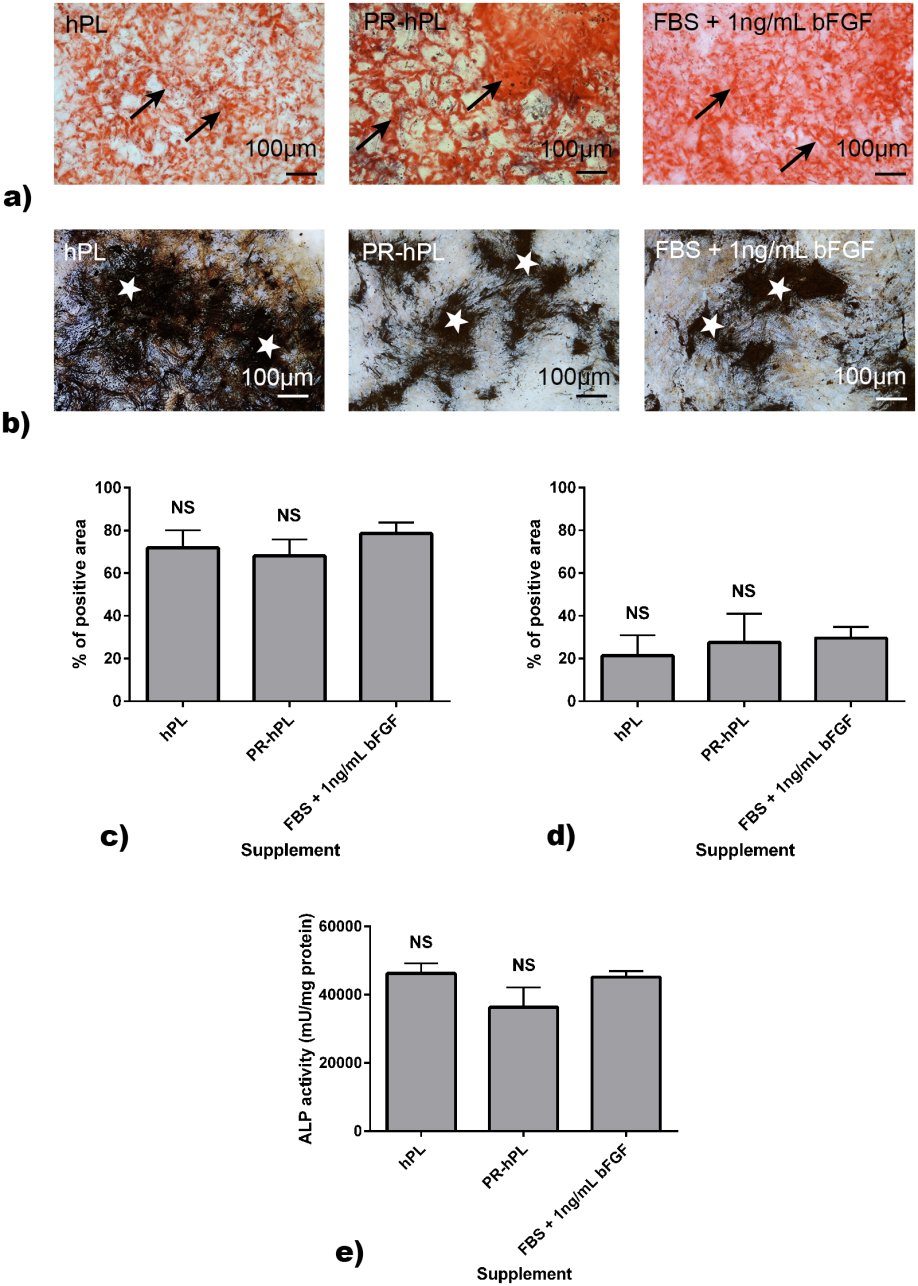
Osteoblast differentiation potential of BM-hMSCs after culture in an FBS+bFGF-, hPL- or PR-hPL-containing medium. Differentiation was induced using the specific medium. The calcium deposit was stained using Alizarin Red S (**a**) and the extracellular matrix using Von Kossa (**b**). Representative photographs of experiments with hMSCs from n = 3 BM. Black arrows and white stars indicated positively stained areas. Quantification of Alizarin Red S (**c**) or Von Kossa (**d**) was expressed as a percentage of positive area. ALP activity measurement was performed using a commercially available kit (**e**). NS: *not significant versus* FBS.

We investigated the effect of the culture conditions on the BM-hMSC multilineage differentiation potential (Figs 6 and 7).

Oil Red O (Fig 6a) and Nile Red (Fig 6b) stainings revealed that BM-hMSCs amplified in an FBS-, hPL-, or PR-hPL-containing medium were able to differentiate in adipocytes. There was no significant difference in the ratios of Nile Red positive cells for BM-hMSCs expanded in hPL or PR-hPL in comparison with FBS (Fig 6c). The quantification of triglycerides showed a significant accumulation after 14 days of differentiation whatever the culture conditions (a level of 5000 RFU was measured before differentiation, Fig 6d).

BM-hMSCs amplified in an hPL- or PR-hPL-containing medium retain their ability to differentiate in osteoblasts, as illustrated with the Alizarin Red S and Von Kossa stainings (Fig 7a and 7b). Quantification of both stainings did not show any significant difference between culture conditions (Fig 7c and 7d). Alizarin Red S, staining calcium-rich deposits [31], displays positivity to a greater degree than Von Kossa. Indeed, the latter is an indirect indicator of calcium, staining phosphate of calcium phosphate [32]. After 21 days of differentiation, the measurement of ALP activity did not show any significant difference between culture conditions (Fig 7e).

Altogether, our results showed that neither hPL nor PR-hPL impaired BM-hMSC differentiation potential in adipocytes and osteoblasts.

### BM-hMSCs cultured in hPL or PR-hPL kept their immunosuppressive properties

**Fig 8:**
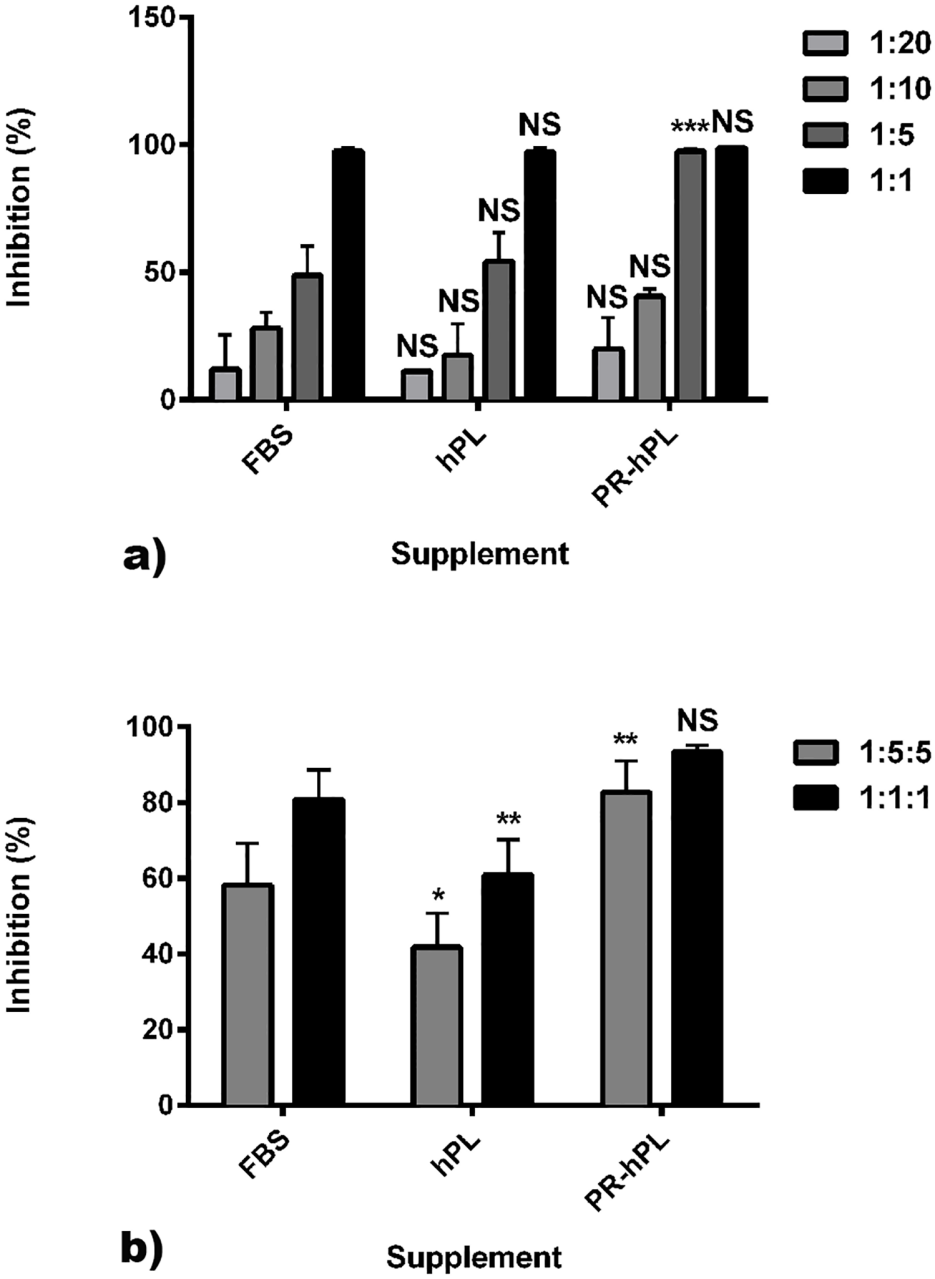
Immunosuppressive properties of BM-hMSCs after culture in an FBS+bFGF-, hPL- or PR-hPL-containing medium. T-cell proliferation was induced using Con A (**a**) or MLR assay (**b**). Results are presented as the percentage of inhibition of T-cell proliferation in experiments performed in quadruplicates. Experiments were performed with MSC:T-cell ratios of 1:20, 1:10, 1:5 and 1:1 (**a**) and MSC:T-cell:PBMC ratios of 1:5:5 and 1:1:1 (**b**) NS: *not significant*, *: *p<0*.*05*; **: *p<0*.*01* and ***: *p<0*.*001 versus* FBS (two-way ANOVA and Bonferroni posttests).

Lastly, we investigated the effect of the culture conditions on the BM-hMSC immunosuppressive properties (Fig 8). T-cell proliferation was induced using Con A and MLR assay. T-cell proliferation was determined after five days using ^3^H-thymidine incorporation. We obtained a 104-fold and 14-fold activation using Con A induction and MLR assay, respectively (66,136 cpm ± 5,868 cpm and 9,250 cpm ± 5,608 cpm, respectively, *versus* 639 ± 414 cpm for non-activated cells).

We observed that mitogen-induced T-cell proliferation was inhibited by BM-hMSCs in a dose-dependent manner, whatever the culture conditions (Fig 8a). At the lowest ratio (1:20), results showed 12.10% ± 13.33%, 11.12% ± 0.61% and 19.94% ± 12.21% of inhibition with BM-hMSCs previously cultured in FBS, hPL and PR-hPL, respectively. We obtained inhibition up to 97.57% ± 0.98%, 97.37% ± 1.48% and 98.84% ± 0.29% with BM-hMSCs previously cultured in FBS, hPL and PR-hPL, respectively (BM-hMSC:T-cell ratio 1:1).

The proliferation of T-cells in MLR assay was inhibited up to 80.91% ± 7.68%, 60.93% ± 9.20% and 93.38% ± 2.17% with BM-hMSCs previously cultured in FBS, hPL and PR-hPL, respectively (BM-hMSC:T-cell:PBMC ratio 1:1:1) (Fig 8b).

Using hPL or PR-hPL for BM-hMSC culture does not alter their immunosuppressive properties.

## Discussion

It has been widely published that hPL is an advantageous substitute for FBS [11–13, 33]. The use of FBS raises xenogenic issues, such as immunological, virological and prion risks. The immunological risk is lowered using human supplements such as hPL; nevertheless, the risk remains that human proteins may be incorporated in infused cells and lead to immunological reactions in the receiving patient. Autologous use may reduce this risk. Concerning the virological risk, hPL is prepared from human blood products obtained from screened (qualified) donors, and every blood collection is tested according to national regulations on blood products. However, zero risk does not exist, and prions are still an issue. Autologous use may reduce contamination risks with viruses and prions. It is well-known that FBS presents batch-to-batch variability, requiring batch screening for hMSC culture, whereas PCs are produced according to standard operating procedures, thus minimizing biological product variation. For this reason, it is possible to standardize hPL, by increasing the batch size via the pooling of several PCs, thus limiting batch-to-batch variability. Neither FBS nor hPL are defined products, but hPL, as a human supplement, can be characterized more easily. Finally, the use of FBS leads to ethical issues, regarding the origin of the product (bovine fetus) and the conditions of production, which are not applicable for hPL.

As previously shown, we confirmed in our study that, in comparison with cells cultured in FBS with bFGF, BM-hMSCs cultured in hPL display a better proliferation rate and fully meet the quality control criteria (expression of membrane markers, potential of adipocytes and osteoblasts differentiation, and inhibition of T-cell proliferation). Moreover, we demonstrated for the first time that the THERAFLEX UV-Platelets procedure performed on PCs has no impact on hPL quality and efficacy. The proliferation of BM-hMSCs in the PR-hPL-containing medium was still better than in the FBS-containing medium, and the quality of expanded BM-hMSCs was maintained (no difference observed in clonogenicity, membrane marker expression, differentiation potential and immunosuppressive properties). The only modification observed was a moderate but significant decrease of hPL TGF-ß1 content without any consequence on hPL quality and efficacy. In addition, the use of PR-hPL can also contribute to viral safety. We consequently demonstrated the feasibility of using THERAFLEX UV-Platelets-treated PCs to subsequently obtain hPL suitable for the scalable efficient expansion of BM-hMSCs in an optimally secured (pathogen-reduced) medium.

We also showed that the supplementation of the medium with heparin at 2IU/mL, a dose that is largely sufficient to avoid the gelation of the medium, resulted in the optimal cell culture conditions for the proliferation of BM-hMSCs. Heparin is available as an authorized drug suitable for injection and is widely used in transfusion medicine. As an anticoagulant, heparin is also currently used for BM aspirate prior to hMSC isolation and consequently may already be part of the process [34]. The use of heparin for hPL-expanded hMSCs intended for infusion in patients has already been reported in clinical trials [35]. Clinical grade heparin production, as an animal-derived biological product, is highly regulated (monographs in the Eur. Ph. and good manufacturing practices [GMP]), from the raw material (mucus) to the final product (purified fractionated heparin) and includes mandatory virus inactivation step(s). However, we demonstrated that an over-dosage of heparin could lead to a progressive decrease in BM-hMSC proliferation. An appropriate dosage of heparin added in the medium is thus necessary to maintain the optimal cell culture conditions.

It has been widely published that hPL is rich in growth-promoting factors. The dosage of growth factors may be a way to characterize such a product. Among them, PDGF-AB [11–13, 33, 36, 37], bFGF [11–13, 33, 36, 37], TGF-ß1 [11, 13, 33, 36, 37], IGF-1 [11, 13, 37], VEGF [11, 12, 33, 36, 37] and EGF [12, 37] are widely described to be present in hPL in large amounts and may represent relevant quality attributes. Produced by the liver, IGF-1 is a major plasmatic growth factor [38] and its level can be correlated with plasma content in hPL. Even if the documentation is poor about which elements in hPL are critical for hMSC proliferation, PDGF [33, 39, 40], bFGF [33, 39, 40], TGF-ß1 [33, 40] and IGF-1 [39] pathways have been suggested to be involved. PDGF-AB, IGF-1 and EGF have been described to promote hMSC migration [2]. VEGF is a key element of the hematopoietic niche, secreted by the hMSCs in the BM [41].

Among the six growth factors we assayed, the only one significantly affected by UV-C illumination is TGF-ß1, with a decrease of 21% (from 61 ± 12 ng/mL to 48 ± 13 ng/mL). TGF-ß1 is the most abundant of the three isoforms of TGF-ß. It acts by binding TGF-ß receptors I and II, leading to Smad2 and Smad3 phosphorylation. Smad4 associated with phosphorylated Smads form a complex that translocates to the nucleus and acts as a transcription factor [42].

First described as a potent chemotactic factor, TGF-ß1 has been shown to display pro- and anti-inflammatory properties [43]. The involvement of TGF-ß1 in the immunosuppressive properties of hMSCs has not been clearly described. More particularly, it has been shown that adding neutralizing TGF-ß1 monoclonal antibody decreases the inhibition of PBMC proliferation by hMSCs [4] but this result has not been reproduced by others [44].

Using neutralizing TGF-ß1 monoclonal antibody, Fekete et al. suggested the involvement of TGF-ß1 in BM-hMSC proliferation [33]. However, TGF-ß1 whether associated or not with other trophic factors, failed to promote BM-hMSC proliferation in the absence of any supplement [33, 39], but a cocktail of factors including TGF-ß1 increased the proliferation in the presence of a small percentage of hPL. These results suggested that TGF-ß1 may be involved in BM-hMSC proliferation, acting synergistically with other factors in hPL. Studies from other groups have shown that TGF-ß1 may increase [40] or inhibit [45] the proliferation of hMSCs from BM or from endometria [46].

In our study, the decrease in TGF-ß1 content of PR-hPL was not correlated with any difference in terms of BM-hMSC proliferation or immunosuppressive properties. On one hand, the results described above are not clear cut and on the other hand, the still high level of TGF-ß1 in PR-hPL suggest that the decrease in TGF-ß1 content between hPL and PR-hPL has no impact on MSC proliferation or immune properties.

In our study, ConA-induced T-cell proliferation inhibition by BM-hMSCs was maintained with the three different cell culture conditions. No significant difference was observed between FBS and hPL. This result was in agreement with other studies that did not observe any significant difference between those two supplements in terms of immunosuppressive properties of BM-hMSCs [12, 13, 47]. However, some studies showed that BM-hMSCs cultured in hPL displayed lesser properties of T-cell proliferation inhibition than BM-hMSCs cultured in FBS [11, 48, 49]. In our study, an increase of T-cell proliferation inhibition was even obtained with PR-hPL but only at the 1:5 ratio.

In MLR assay, we observed some moderate but significant differences between the different cell culture conditions. The percentage of inhibition of T-cell proliferation was decreased when BM-hMSCs were cultured in hPL in comparison with FBS and increased when BM-hMSCs were cultured in PR-hPL. However, this increase of inhibition was found significant with the 1:5:5 ratio only.

Altogether, our results showed that the immunosuppressive properties of hMSCs were maintained whatever the cell culture conditions. The differences observed between the ConA-induction and the MLR assay must be interpreted carefully and put back in perspective with the works of Capelli and collaborators that interestingly showed that the method used (mitogen-induced T-cell proliferation or MLR assay) may induce some variations in the results [50].

The significant differences found in this study suggest that PR-hPL could represent a better medium supplement for BM-hMSCs in terms of the inhibition of T-cell proliferation. We could make the hypothesis that the alteration by the UV-C treatment of factors (that need to be identified) potentially involved in the immunomodulatory mechanisms could explain, at least in part, the increase of T-cell proliferation inhibition obtained when BM-hMSCs were cultured in PR-hPL.

Nevertheless, because the immunomodulation properties of cells result from multi-parameter and complex factors and, as described by others, results can differ depending on the method used or the cell history [50, 51], a specific study would be needed in order to identify the potential factors that could be involved and to better understand the potential impact of PR-hPL on the optimization of BM-hMSC immunosuppressive properties.

Three technologies have been developed for pathogen reduction in PCs [15, 20]. Intercept technology (described in [52]) is based on the blockage of pathogen replication by a synthetic psoralen (Amotosalen, S-59). Upon UV-A illumination (320–400nm), S-59 induces irreversible damage in nucleic acids. The Mirasol technology associates and combines the damaging effects of vitamin B2 (riboflavin) and UV-A and -B illumination (285–365nm) on nucleic acids [53]. The THERAFLEX UV-Platelets technology (fully described in [23]) is based on short-wave UV illumination (254 nm) without the need for any additive. At this wavelength, UV-C light generates damage specifically in nucleic acids that is too extensive to be reversible. The THERAFLEX UV-Platelets procedure is currently validated for PCs prepared in the platelet additive solution SSP+. The efficacy of these three technologies against bacteria [54–56] and enveloped and non-enveloped viruses [26, 56, 57] has been described. The THERAFLEX UV-Platelets technology is particularly effective against hepatitis C, Influenza A and Chikungunya viruses (≥ 5.0, ≥ 5.3 and 6.34 log-reduction, respectively) [20, 58]. None of these technologies is reported to be effective on all types of viruses. For example, HIV-1 is only moderately reduced by THERAFLEX UV-Platelets, hepatitis A virus inactivation is not effective with Intercept [59] and Dengue viruses are slightly reduced following treatment with riboflavin and UV light [60]. In addition, the three technologies have been shown to perform inactivation of residual leukocytes [61–63], thus lowering the risk of transfusion-associated graft-versus-host disease. The preservation of platelets quality has been described for the three technologies, with only a slight activation of the cells and a slight increase of their metabolism [55, 64, 65].

In contrast to UV-A- or UV-B-based technologies, the THERAFLEX UV-Platelets technology does not need any photosensitizing additive. As a consequence, any risk of adverse effects with the photochemicals, their photoproducts or impurities (immune reactions, toxicity and carcinogenicity) can be excluded. Indeed, it has been estimated that residual quantities of S-59 (1 μg/kg) and its photoproducts (115 μg/kg) have been infused to the platelet recipient after pathogen reduction using Intercept technology [52]. Even though toxicological studies in animals [52, 66] and clinical trials [67–70] have demonstrated the safety of photochemical-based pathogen reduction technologies, long-term studies may be needed for the evaluation of the risk of adverse effects, especially of carcinogenesis [23].

In one hand, it has been described that hPL prepared from Intercept-inactivated PCs is equally good as non-inactivated hPL as a supplement for BM-hMSC proliferation [36, 71, 72]. On the other hand, it has been difficult to assess the safety margin of psoralen or its photoproducts bound to or incorporated by platelets when transfused into a patient [23] and quantities infused into the recipient may be increased in the case of BM-hMSCs cultured for several passages in hPL prepared from pathogen-reduced PCs. Considering this and the fact that pathogen-reduced PCs using S-59 and UV-A are contraindicated in patients displaying allergy to psoralens, the use of hPL prepared from pathogen-reduced PCs using Intercept technology [36, 71, 72] for BM-hMSCs intended for clinical use may lead to a regulatory issue. In this context, an additive-free technology, consequently with no need for conventional pharmacokinetic and toxicological assessments [20], may remain advantageous.

Our results demonstrate for the first time the feasibility of using the additive-free THERAFLEX UV-Platelets technology to subsequently obtain a pathogen-reduced hPL, suitable for GMP-compliant proliferation of BM-hMSCs intended for clinical use.

## Supporting information

S1 Fig Impact of heparin concentration on the proliferation of BM-hMSCs cultured in an FBS+bFGF- or hPL-containing medium. BM-hMSCs were cultured for 7 days in a 10% FBS + 1ng/mL bFGF- (**a**) or 8% hPL- (**b**) containing medium. Heparin was added at doses ranging from 0 to 64 IU/mL (**a**) or 1 to 64 IU/mL (**b**). Results are presented as a proliferation ratio to the lowest heparin concentration for means of triplicates. NS: *not significant*; *: *p<0*.*05*; ***: *p<0*.*001 versus* the lowest heparin concentration (one-way ANOVA and Bonferroni posttests).
(DOCX)
