# Supplementary material for: Pathogen reduction through additive-free short-wave UV light irradiation retains the optimal efficacy of human platelet lysate for the expansion of human bone marrow mesenchymal stem cells
Source: PLoS One. 2017 Aug 1;12(8):e0181406. doi: 10.1371/journal.pone.0181406 (PMC5538655; doi:10.1371/journal.pone.0181406)
Supplement: S1 Fig — BM-hMSCs were cultured for 7 days in a 10% FBS + 1ng/mL bFGF- (a) or 8% hPL- (b) containing medium. Heparin was added at doses ranging from 0 to 64 IU/mL (a) or 1 to 64 IU/mL (b). Results are presented as a proliferation ratio to the lowest heparin concentration for means of triplicates. NS: not significant; *: p<0.05; ***: p<0.001 versus the lowest heparin concentration (one-way ANOVA and Bonferroni posttests). (DOCX) [file pone.0181406.s001.docx]

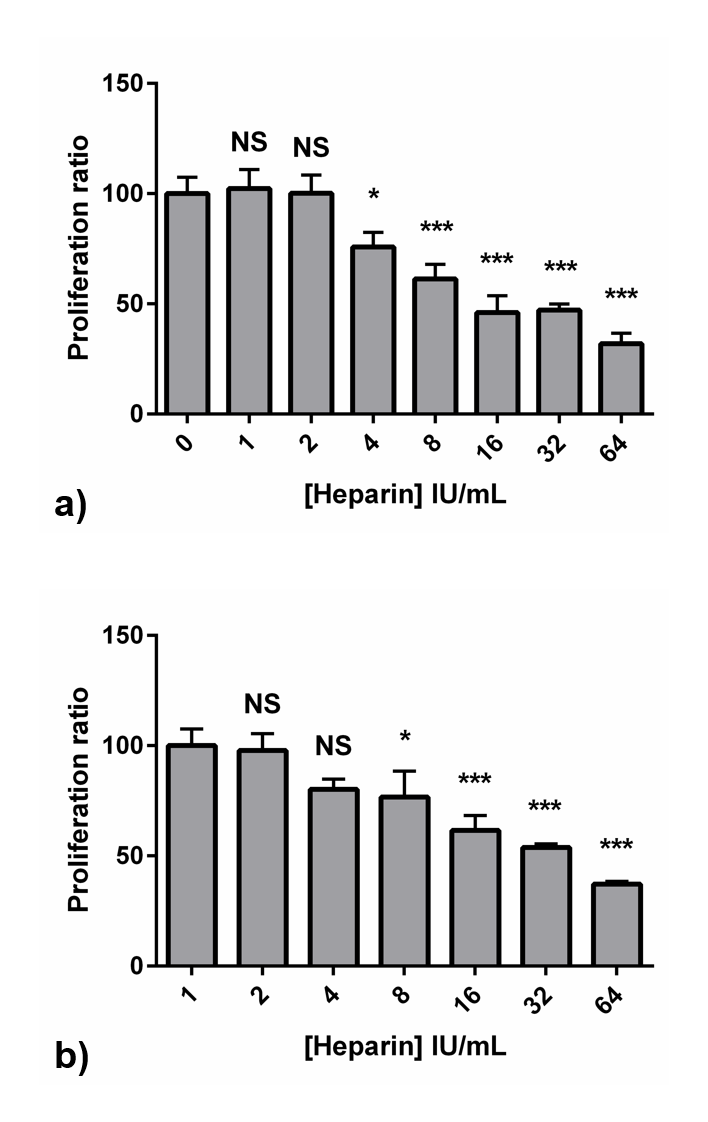


**S1 Fig: Impact of heparin concentration on the proliferation of BM-hMSCs cultured in an FBS+bFGF- or hPL-containing medium.** BM-hMSCs were cultured for 7 days in a 10% FBS + 1ng/mL bFGF- (**a**) or 8% hPL- (**b**) containing medium. Heparin was added at doses ranging from 0 to 64 IU/mL (**a**) or 1 to 64 IU/mL (**b**). Results are presented as a proliferation ratio to the lowest heparin concentration for means of triplicates. NS: *not significant*; *: *p<0.05*; ***: *p<0.001* *versus* the lowest heparin concentration (one-way ANOVA and Bonferroni posttests).
